# A Comparative Study on Predication of Appropriate Mechanical Ventilation Mode through Machine Learning Approach

**DOI:** 10.3390/bioengineering10040418

**Published:** 2023-03-27

**Authors:** Jayant Giri, Hamad A. Al-Lohedan, Faruq Mohammad, Ahmed A. Soleiman, Rajkumar Chadge, Chetan Mahatme, Neeraj Sunheriya, Pallavi Giri, Dhananjay Mutyarapwar, Shreya Dhapke

**Affiliations:** 1 Mechanical Department, Yeshwantrao Chavan College of Engineering, Nagpur 441110, India; 2 Department of Chemistry, College of Science, King Saud University, Riyadh 11451, Saudi Arabia; 3 Department of Chemistry, College of Science, Southern University and A&M College, Baton Rouge, LA 70813, USA; 4 Laxminarayan Institute of Technology, Nagpur 440033, India; 5 Tata Consultancy Services, Nagpur 441108, India; 6 Cognizant, Hyderabad 500019, India

**Keywords:** mechanical ventilation (MV), machine learning (ML), ventilation mode (VM), optimization, intensive care unit (ICU)

## Abstract

Ventilation mode is one of the most crucial ventilator settings, selected and set by knowledgeable critical care therapists in a critical care unit. The application of a particular ventilation mode must be patient-specific and patient-interactive. The main aim of this study is to provide a detailed outline regarding ventilation mode settings and determine the best machine learning method to create a deployable model for the appropriate selection of ventilation mode on a per breath basis. Per-breath patient data is utilized, preprocessed and finally a data frame is created consisting of five feature columns (inspiratory and expiratory tidal volume, minimum pressure, positive end-expiratory pressure, and previous positive end-expiratory pressure) and one output column (output column consisted of modes to be predicted). The data frame has been split into training and testing datasets with a test size of 30%. Six machine learning algorithms were trained and compared for performance, based on the accuracy, F1 score, sensitivity, and precision. The output shows that the Random-Forest Algorithm was the most precise and accurate in predicting all ventilation modes correctly, out of the all the machine learning algorithms trained. Thus, the Random-Forest machine learning technique can be utilized for predicting optimal ventilation mode setting, if it is properly trained with the help of the most relevant data. Aside from ventilation mode, control parameter settings, alarm settings and other settings may also be adjusted for the mechanical ventilation process utilizing appropriate machine learning, particularly deep learning approaches.

## 1. Introduction

**Figure 1:**
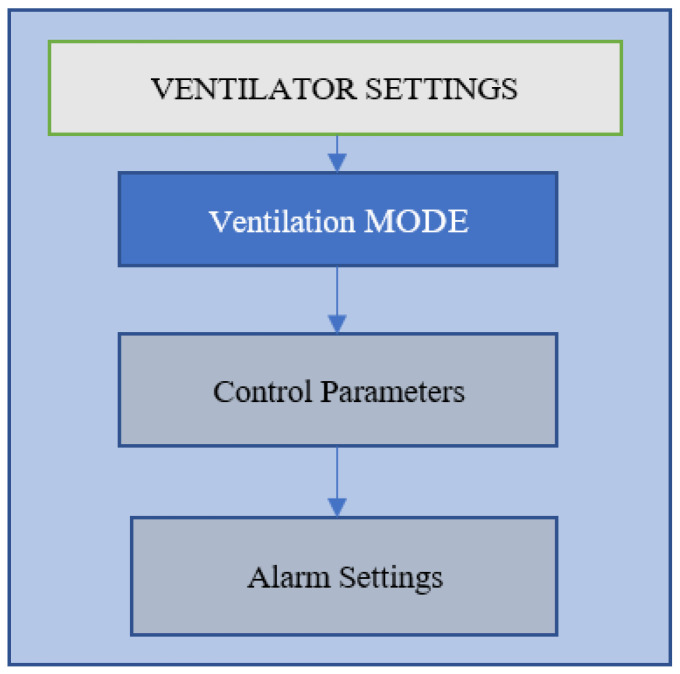
Types of ventilator settings applied in a shown sequence.

One of the most important treatments routinely used in patients with respiratory conditions or disorders is mechanical ventilation (MV). It is a complicated form of therapy that requires quick and precise decision-making. A sub-optimal mechanical ventilation strategy has a chance of creating ventilator-induced lung injury (VILI). The sub-optimality of an individualized ventilation strategy also leads to an increased ICU stay, a higher cost of ventilation, and an elevated risk of death in patients. The complete optimization of the MV process is mainly dependent on three factors: appropriate mode selection, appropriate control parameter settings and alarm settings, as shown in Figure 1. Most clinicians try to avoid intubating patients and prefer to support the patient by the non-invasive ventilation method. However, if the situation is very critical (blocked airways), intubating the patient is necessary. After intubation, the first step is to select an appropriate ventilation mode. Thus, ventilation mode is one of the most important ventilator settings [1]. The ventilator interprets the user’s instructions and then produces a series of breaths with the right volume, flow and pressure of air and oxygen. A selection of preconfigured breath types and sequences are determined by a certain VM setting. Each mode has a set of intended applications. The main characteristics of defined mechanical breaths are determined by control parameter choices.

### 1.1. Ventilation Modes: Origin to Today

Negative pressure ventilation mechanical ventilators first appeared in the early 1800s, and positive pressure ventilation ventilators started to appear in the early 1900s. The development of mechanical ventilators has been the work of numerous researchers [2]. Mechanical ventilators were first incorporated in hospitals on a large scale in the 1960s. It consisted of only the pressure control mode. The ventilator was useful for normalizing the blood–gas amount using pressure control, but it had high injury rates during the treatment of patients with acute respiratory distress syndrome, which requires controlling the amount of tidal volume (delivery of air and oxygen mixture at a set volume) and minute ventilation. As a result, volume control modes for ventilators have been available since the 1970s [3]. The two modalities are interdependent. Pressure data are tracked when the volume is controlled, and vice versa. Both approaches have been used for critical care treatment since the 1980s in accordance with respiratory needs [3]. Traditional ventilators worked well when the patient was in a passive condition. Once the patient became active or partially active, asynchrony was created between the patient and the ventilator. Thus, the clinicians at that time did not have many options when the patient got into a partially active or active condition. Thus, the ventilator industry constantly introduced new modes. All modes were not used in a real-time situation. Today, there are numerous modes available as per patient need. According to Chatburn, a top mechanical ventilation expert, there are a total of 174 unique names for ventilation modes. This was the result of technological advancements and a non-standard nomenclature for naming modes [4]. Since the names of ventilation modes are not defined globally, any manufacturer is allowed to use them. Understanding the distinction between unique modes and unique mode names is crucial. Although there may be 174 unique mode names, there are fewer than 20 unique modes. All contemporary ventilators provide ventilation mode and control parameter settings by default. This capability is extremely beneficial when a patient requires emergency mechanical ventilation while clinicians are occupied with other patients. In these situations, a machine learning model could be quite effective in predicting the modes according to a patient’s changing respiratory demands after the adoption of a default ventilation mode. In the next section, unique modes of ventilation are briefly discussed.

### 1.2. Unique Modes of Mechanical Ventilation

**Figure 2:**
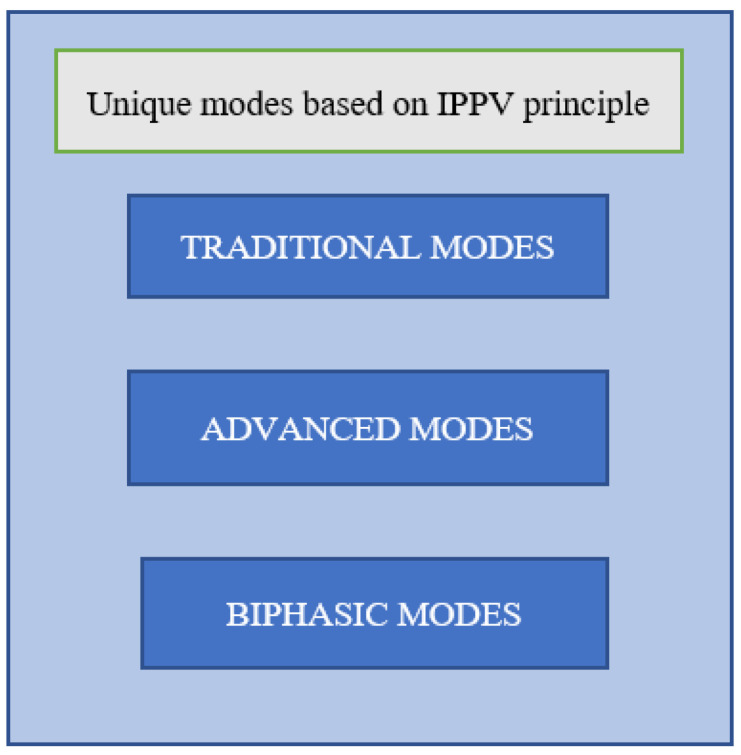
Major classification of ventilation modes.

Although there are 174 distinct mode names due to non-standardized mode nomenclature, all current ventilators have one thing in common: they are based on intermittent positive pressure ventilation (IPPV). The IPPV principle can be conceptualized as a series of forced mechanical breaths supplied by a ventilator system [5]. Based on the IPPV principle, all modes of ventilation fall mainly into one of these three categories, as shown in Figure 2. The first category is that of traditional modes. It consists of eight unique modes based on various parameters. The second category belongs to advanced modes that are the results of consistent growth in technology. In the third category, biphasic modes are included. They supply ventilation breaths by adjusting the positive end-expiratory pressure (PEEP) in a consistent time pattern. Traditional modes are uncomplicated and the most advantageous for real-time applications. These modes are available on the vast majority of mechanical ventilators. Advanced modes and traditional modes have similar breath types. Yet, advanced modes have much more complicated controlling algorithms. They mainly improve treatment quality and decrease the clinical workload. They automate the process of mode adjustment as per the patient’s respiratory needs. They are present in some modern mechanical ventilators. Biphasic mechanisms underpin biphasic modes. The biphasic mechanism is an expansion of the mechanism that generates PEEP. Mechanical breaths can also be generated by the PEEP-generating mechanism [5].

Table 1 represents the classification of modes. It helps identify the unique modes out of the 174 unique mode names. Based on pressure, volume and adaptive control, there are a total of eight traditional modes. Understanding traditional modes helps to give a clear idea of the importance of modes and the differences in the functionality of each mode. The most commonly used modes are the pressure control (P-CMV) mode, volume control (V-CMV) mode, pressure and volume control (SIMV) mode and pressure support mode (PS). Out of the advanced modes, the proportional assist ventilation (PAV) mode and adaptive support ventilation (ASV) mode are mostly used. Biphasic modes are not much, but CPAP and APRV modes are the most commonly used modes among them.

### 1.3. Ventilation Mode: Control Parameters

A patient–ventilator asynchrony is less likely to occur if the ventilation mode setting is correct, patient-specific, patient-interactive and based on the patient’s needs. This enables the patient to recover as soon as possible. This culminates in a shorter duration of stay and lower costs associated with ventilation. There are eight distinct sorts of breaths, each with its own control, triggering, cycling and application settings. The performance of a ventilator system varies based on the types of breath and sequence. Thus, a ventilator system provides a variety of modes to accommodate the unique respiratory needs of each patient. Typically, just one ventilation mode is selected for a given duration. When a respiratory therapist sets a certain mode, it is to be regarded as the mode that best meets the individual’s breathing support requirements. However, as the decision-making process is performed by humans, it is subjected to some degree of suboptimality. Additionally, understanding an individual’s respiratory needs and lung pathology is a complex process. These complications influence the mortality of a patient and may induce morbidities [6,7]. It necessitates a thorough review of patient-related data, which can be complicated, time-consuming and prone to human error. Because of continual technological developments in the hardware development industry, large-scale data gathering and storage are now easily feasible. A vast amount of historical data can be used to train different machine learning (ML) algorithms and to identify distinctive patterns and relationships between data in order to optimize different systems and procedures [8,9].

Table 2 represents the control parameters that are set for a particular mode, such as tidal volume in volume control mode or pressure control in pressure control mode.

Health care institutions generally use MV therapy in their intensive care unit (ICU) to provide care to critically ill patients. It is a rich source of patient data and patient responses to a particular MV strategy [10]. The decision on an optimal ventilation strategy depends upon several factors, such as laboratory data, comorbidities in the patient, vitals, severity of illness scores, disease progression, etc. [11]. Several studies have examined lung protective ventilation strategies, with proper mechanical ventilation mode selection. Mechanical ventilation is primarily used to reduce the risk of ventilator-induced lung injury (VILI) in patients with acute respiratory distress syndrome (ARDS). VILI is a condition that can arise in mechanically ventilated patients as a result of a combination of excessive pressure (barotrauma), excessive volume (volutrauma), repeated alveolar opening and shutting (atelectrauma), inflammatory mediator release from the lung (biotrauma) and oxygen toxicity [12,13,14,15,16,17,18,19,20]. The personalization of mechanical breathing based on individual physiological parameters and therapeutic responses can enhance clinical outcomes [21].

Regarding the creation of machine learning models in conjunction with mechanical ventilation and its optimization, a number of unique works have been assessed. Deep learning (DL) methods such as convolutional neural networks (CNN) and long short-term memory (LSTM) neural networks find their application in analyzing several unique features of ventilator waveforms. During mechanical ventilation, three types of waveforms are generated: an inspiratory–expiratory pressure waveform, an inspiratory–expiratory flow of air–gas mixture waveform and an inspiratory–expiratory volume of air–gas mixture waveform. A CNN DL model was developed to detect patient–ventilator asynchrony (PVA) events [22]. This model only detected the PVA events but could not classify the type of PVA that occurred. As a result, an LSTM DL model was created to identify and categorize the different types of patient–ventilator asynchrony [23]. These models excluded the need for continuous monitoring of a patient’s condition. A Random-Forest ML model [24] was also developed to achieve similar goals. A model for asynchrony detection would not be essential, though, provided the ventilator settings are optimal, patient-specific and interactive. A gradient boosting ML model was developed based on the severity of illness scores to predict whether a patient was at risk of prolonged mechanical ventilation or tracheostomy placement [25]. An XGBoost ML model was developed for predicting patients at risk for sepsis to ensure timely diagnosis before the situation gets out of control [26]. A regression Decision-Tree ML model was developed to predict resistance (R) and compliance (C) values. A set of airway pressure, flow rate and their respective R and C values were used for the creation of the model [27]. A LightGBM ML model was developed to predict extubation success or failure before starting the execution of spontaneous breathing trials [28]. A similar model was developed using a support vector machine (SVM) algorithm for predicting extubation success or failure [29]. The difference between both models was that the support vector machine model was more accurate than the LightGBM model. These models were useful for preventing the patients from premature extubation (re-intubation) and prolonged extubation. Similar research was also conducted on the accurate prediction of the respiratory rate and blood oxygen level using PPG signals with the help of a machine learning model [30]. BubbleVent, a mechanical ventilator, was manufactured and tested for effective mechanical ventilation and was able to deliver stable PIP and PEEP levels [31]. For accurate monitoring of respiratory mechanics, a study was successfully completed on a model-based assessment of asynchrony events for mechanically ventilated patients [32]. A machine learning model was applied and tested with success for parameter estimation for mechanical ventilation [33]. A rule-based model was developed for predicting the modes of ventilation on a per-hour basis [34,35]. An ML model was also developed to predict the modes of ventilation on a per breath basis. The analysis was performed for only one VM while keeping other VMs constant [36]. Thus, after going through several pieces of literature, it was found that optimization of the ventilation mode setting using machine learning is still a promising area. The goal of this study is to create a new data frame using the best feature selection test. The optimum machine learning approach for a ventilation mode prediction on a per-breath basis was then determined by fitting the data frame over six distinct machine learning techniques. Based on the various ML algorithms’ accuracy, sensitivity, precision and F1 score for predicting VM, a comparison analysis is carried out. Section 2 discusses the data collecting and preprocessing steps required to produce a final data frame, and the results and discussion section compares various ML algorithms fitted on the data frame. The outcome of the current investigation is presented in Section 4.

## 2. Material and Methods

This section provides an overview of the dataset type, data collection method and data preprocessing procedure employed in the current investigation. Additionally, a descriptive and reliable statistical analysis of the data is provided.

### 2.1. Data Collection

Data collection is the most important part of the creation of an ML model. For this study, the data was referred from the existing literature [36]. The data consisted of several data files having almost 2000–4000 rows of the patient’s breath data. Each row represented a single breath. A specific and appropriate VM was given to each breath. Annotations of each breath were conducted by a group of three respiratory experts mentioned in the literature [36]. Each row of data file consisted of information (for a single breath) about tidal volume (both inspiratory and expiratory) and their ratio (expiratory to inspiratory), minimum pressure (Pmin), value of positive end-expiratory pressure in the previous breath (PEEPprev), inspiratory time and expiratory time, positive end-expiratory pressure (PEEP) value, etc. Thus, the data in each data file were continuous, which was necessary for the creation of the mode prediction model as VM is a continuous setting. It does not change unless an operator intervenes. Out of the five available modes, any one will be the output as per the respective input features. Those five modes were: pressure control mode (PC), volume control mode (VC), pressure support mode (PS), pressure-assist ventilation (PAV) mode and continuous positive airway pressure (CPAP) mode. PC, VC and PS are the traditional modes of ventilation. These are the most basic and popular modes. They can be found in the majority of ventilators. PAV is a sophisticated mode that was created using a complex controlling algorithm. Most modern ventilators operate in this mode. Most contemporary ventilators use the biphasic mode called CPAP. This study includes these three categories of distinctive modes.

### 2.2. Data Preprocessing

**Figure 3:**
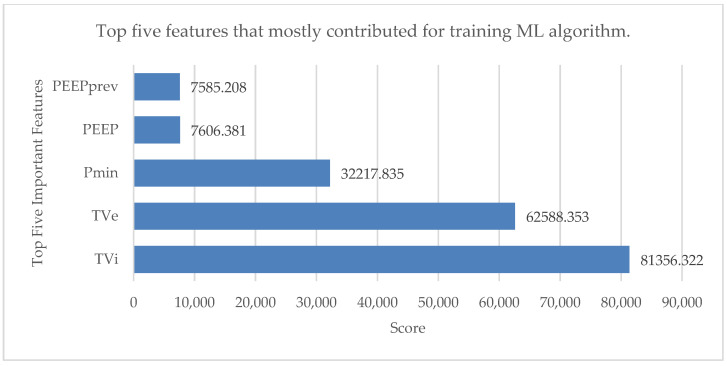
Top five features that mostly contributed for training ML algorithm.

Preprocessing the data is essential to the development of ML models. The monitoring and therapeutic technologies used in critical care units (such as mechanical ventilators) continuously produce significant amounts of data, making these settings particularly data-rich. The most pertinent data attributes are chosen rather than using the entire set of data to create the model. A feature selection test was performed to get the top five features that contributed significantly to the training of the ML algorithm. This small subset of features from the available dataset is significant for the analysis [37]. The results are shown in Figure 3 with the help of a horizontal bar chart.

Those five features were inspiratory tidal volume (TVi), expiratory tidal volume (TVe), PEEPprev value, minimum pressure value (Pmin) and positive end-expiratory pressure (PEEP). All the data files were segregated, and each row was annotated as one of these five modes: pressure control mode (PC), volume control mode (VC), pressure support mode (PS), pressure-assist ventilation (PAV) mode and continuous positive airway pressure (CPAP) mode. Missing data and rows with negative values were deleted from the data files for the efficient handling of the data. The top five features were finally combined into a single data frame because the remaining features (tidal volume expiratory to inspiratory ratio, expiratory to inspiratory ratio and inspiratory to expiratory duration) had very little effect on training the ML algorithms. Five feature columns and one output column made up the final data frame. The single-breath data were organized in 20,237 rows.

**Figure 4:**
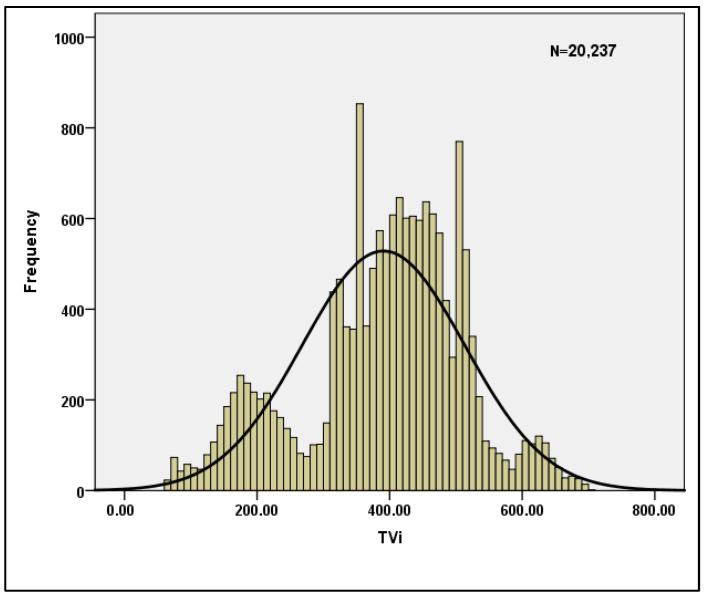
Normality plot for TVi.

**Figure 5:**
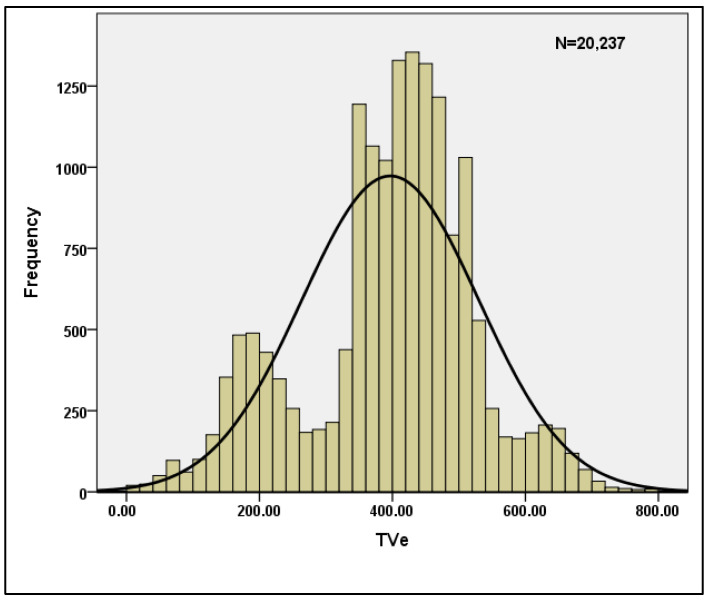
Normality plot for TVe.

Table 3 shows the descriptive statistics for the per breath data when n = 20,237 is used. The skewness and Kurtosis values shown in the table clearly indicate that the data are normal, and hence the data are suitable for machine learning models. Table 4 displays the reliability statistics, with a Cronbach’s Alpha score of 0.787 indicating that the data are reliable. With a conditional probability value of 0, the Hotelling’s T-squared test is statistically significant. Furthermore, for the sample characteristics TVi and TVe, the normality of the data is plotted and shown in Figure 4 and Figure 5.

Table 5 depicts the Pearson co-relationship for input variables, and it is clear that multi-collinearity does not exist, indicating that the inputs can be used as independent variables in machine learning. The data frame was split into training and testing datasets, with a test size of 30% of the data frame. Based on the training dataset, the data were fitted on six different ML algorithms. Those six ML algorithms included: Random-Forest (RFC), Logistic Regression (LR), Gaussian Naive Bayes (GNB), LinearSVC (SVM), KNeighbors (KNN) and Decision-Tree (DTREE). The projected output was compared to the desired output using the test features dataset (original test output). The performance of each method was evaluated using evaluation reports which included precision, sensitivity, F1 score and accuracy numbers. Section 3 includes a comparison analysis that compares the performance of various ML algorithms in predicting each mode of ventilation.

The parameters tuned into the ML algorithms during the model fitting procedure are shown in Table 6. Parameters are critical to the correct training of an ML algorithm. The performance of the final ML model improves when the correct parameters are tuned during the model fitting process.

## 3. Results and Discussion

Evaluation reports for each algorithm were acquired based on algorithm training and testing, and are provided in Table 7, Table 8, Table 9, Table 10, Table 11 and Table 12, respectively. Six different ML algorithms were employed. In this work, ML algorithms were utilized to determine the difference in their performance when trained and tested on the same data frame. It also aided in determining which ML algorithms are appropriate for use in the mechanical ventilation domain in order to construct more accurate models based on the type of data provided. On a per-breath basis, the model was only required to predict one of the five potential modes. The five modes were pressure control (PC), volume control (VC), pressure support (PS), proportional assist ventilation (PAV) and continuous positive airway pressure (CPAP).

**Figure 6:**
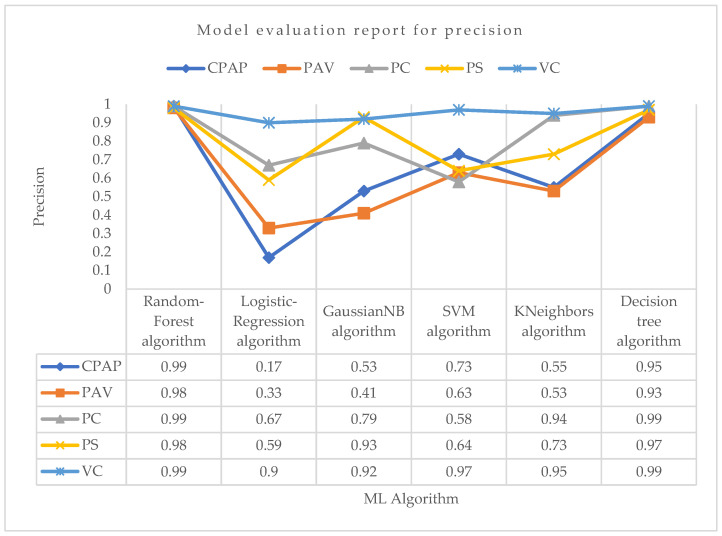
Model evaluation report of precision for different modes and ML algorithms.

Using the precision parameter shown in Figure 6 for PC mode, the Random-Forest and Decision-Tree algorithms achieved the highest precision of 0.99. Aside from these two techniques, the KNeighbors algorithm had the second-highest precision in predicting PC mode. Other algorithms did not perform well compared with them. Again, the Random-Forest and Decision-Tree algorithms achieved the maximum precision in VC mode, which was around 0.99. The support vector machine algorithm achieved the second-highest precision, which was around 0.97. KNeighbors performed well, although it was not the most accurate in predicting VC mode. For PS mode, the highest precision was obtained by the Random-Forest algorithm, which was about 0.98. The Decision-Tree was the second highest and GaussianNB was the third most precise in correctly predicting PS mode, with precision values of about 0.97 and 0.93, respectively. The Random-Forest method achieved the highest precision in PAV mode, at roughly 0.98, followed by the Decision-Tree technique, at 0.93. With a precision score of 0.99 for CPAP mode, Random-Forest was the most successful method. With the least precision value of 0.17 in predicting CPAP mode, the logistic regression did not perform well.

**Figure 7:**
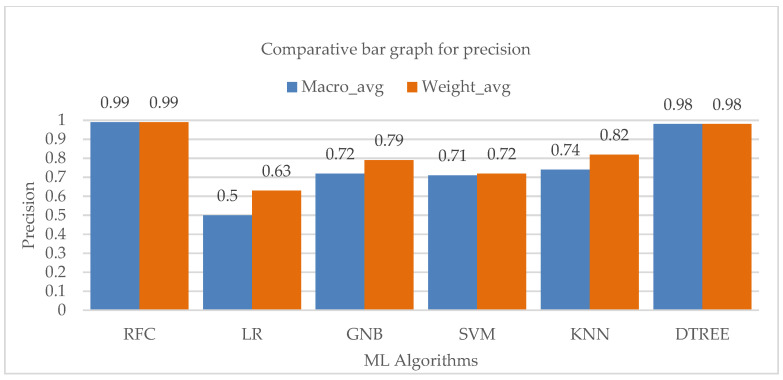
Bar chart visualization of precision comparison among different ML algorithms.

The macro and weighted average of the accuracy values of the various machine learning techniques is shown in Figure 7, which also demonstrates a considerable contribution of the Random-Forest and Decision-Tree algorithms to the overall precision of the entire class.

**Figure 8:**
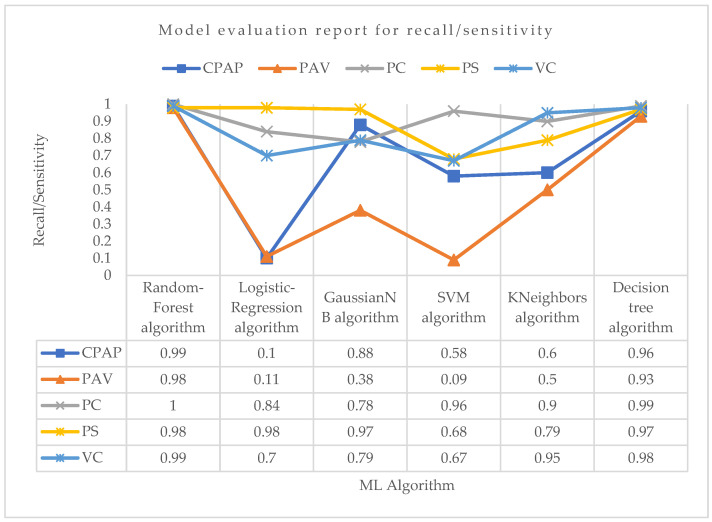
Model evaluation report of recall/sensitivity for different modes and ML algorithms.

Now, considering the recall, i.e., sensitivity parameter, for PC mode as shown in Figure 8, Random-Forest outperformed all the algorithms with the highest recall of 1.0. For VC mode, the Random-Forest and Decision-Tree algorithms both performed well in terms of recall, with a value of 0.99 and 0.98, respectively. For PS mode, the logistic regression and Random-Forest algorithms had the highest recall value of about 0.98, followed by the Decision-Tree algorithm with a recall value of 0.97. With a recall value of 0.98 for PAV mode, the Random-Forest algorithm surpassed all other ML algorithms. With recall values of 0.09 and 0.11, respectively, support vector machine and logistic regression had the weakest recall performance for properly predicting PAV mode. The Random-Forest algorithm showed the maximum recall in the CPAP mode, at roughly 0.99. Although they both performed well, the Decision-Tree and GaussianNB algorithms were not the best at accurately predicting CPAP mode. Again, the lowest recall score for accurately predicting CPAP mode was seen with the logistic regression.

**Figure 9:**
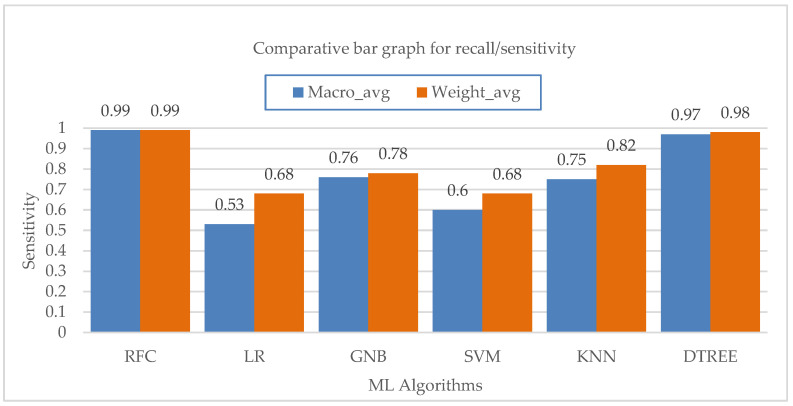
Bar chart visualization of sensitivity comparison among different ML algorithms.

**Figure 10:**
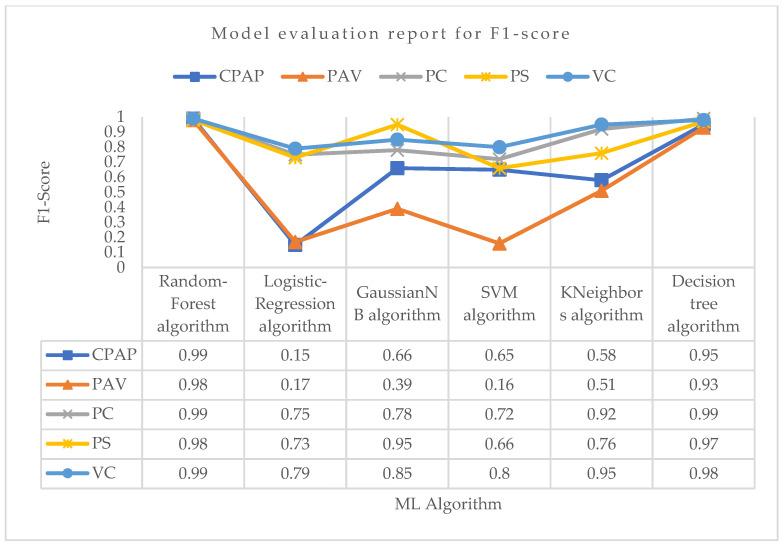
Model evaluation report of F1 score for different modes and ML algorithms.

Figure 9 shows the average values for the sensitivity of different machine learning algorithms at the macro and weighted levels. The Random-Forest and Decision-Tree algorithms both have a major impact on the overall sensitivity of the class. Lastly, considering the F1 score parameter for PC mode as shown in Figure 10, the Random-Forest and Decision-Tree algorithms had the highest F1 score of about 0.99. For VC mode, the Random-Forest algorithm had the highest F1 score of about 0.99, followed by the Decision-Tree algorithm with an F1 score value of 0.98. For PS mode, the Random-Forest algorithm had the highest F1 score value of about 0.98, followed by the Decision-Tree algorithm with a score of 0.97. For PAV mode, the Random-Forest algorithm had the highest F1 score of 0.98, followed by the Decision-Tree algorithm with an F1 score of 0.93. The rest of the algorithms did not perform well in terms of F1 score in correctly predicting PAV mode. Lastly, for CPAP mode, the highest F1 score was achieved by the Random-Forest algorithm with a value of 0.99, followed by the Decision-Tree algorithm with a value of 0.95. The logistic regression did not perform well in correctly predicting CPAP mode with the least F1 score value of 0.15.

**Figure 11:**
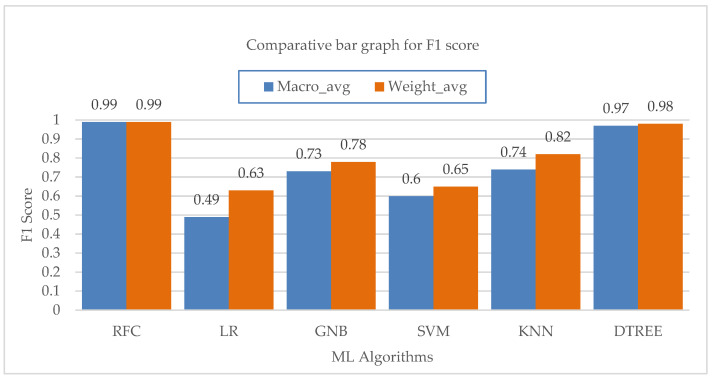
Bar chart visualization of F1 score comparison among different ML algorithms.

**Figure 12:**
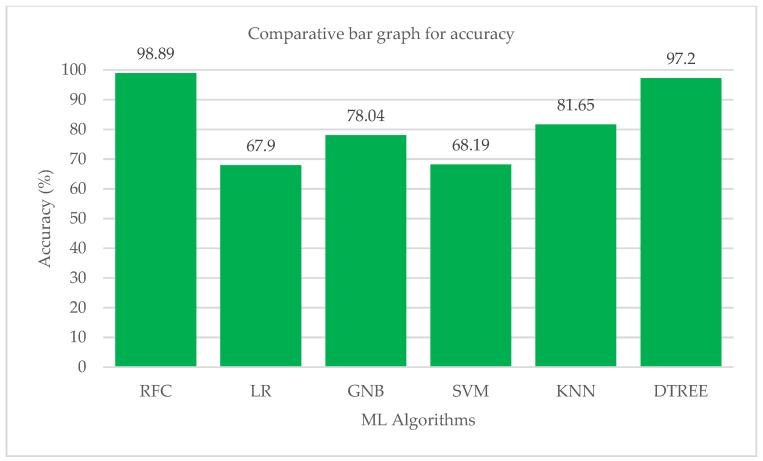
Bar chart visualization of accuracy comparison among different ML algorithms.

The macro and weighted average values of the F1 score for various machine learning algorithms are shown in Figure 11. The KNN method is followed by the Random-Forest and Decision-Tree algorithms in terms of their effects on the class’s total F1-score. According to performance statistics, the bar chart visualization and a comparison analysis, it is clear that the Decision-Tree and Random-Forest algorithms are the most effective in accurately predicting all five modes with substantial and high values of precision, recall and F1 score. The accuracy values of each ML algorithm are shown in Table 13. Figure 12 shows a bar chart representation of the various accuracy levels attained by several ML algorithms employed in per-breath prediction modes.

The Random-Forest algorithm correctly and precisely predicted all of the ventilation modes that were included in the study. After Random-Forest, the Decision-Tree method has demonstrated an impressive performance in predicting all five modes with high precision, sensitivity and accuracy values. The KNeighbors algorithm performed brilliantly in terms of accuracy, but it was less skilled at precisely predicting each mode. Only the Random-Forest algorithm had successfully predicted each mode of ventilation with very good sensitivity and precision. The high precision, sensitivity and accuracy of each mode’s prediction differed between the logistic regression, support vector machine and Gaussian Naive Bayes algorithms. Cross-validations were performed using a 10k-fold consistency evaluation to get the best performance of all the ML models in the study. The Random-Forest algorithm successfully predicted each mode of ventilation properly each time, with high values for precision, sensitivity, F1 score and accuracy for the present investigation. The Random-Forest technique correctly predicted each mode with a performance between 0.98 and 1.00 in terms of precision, sensitivity, F1 score and accuracy. The other modes, however, have variations in their accuracy, sensitivity, F1 score, and precision. The precision, sensitivity, and F1 score values for each mode varied greatly after the algorithms for logistic regression, Gaussian Naive Bayes and support vector machines were trained and assessed. The Decision-Tree and KNeighbors algorithms consistently performed well in correctly forecasting each mode, but their performance did not improve significantly as compared to the Random-Forest model. According to the comparative study’s findings, the ability of the logistic regression to accurately predict the CPAP mode was subpar. It fared mediocrely in all other modes. With a recall score of 0.98, it did an excellent job at predicting PS mode. In the mode of predicting PAV, the performance of the support vector machine method was the worst. Moreover, the logistic regression also struggled to accurately identify the PAV mode. In terms of forecasting all modes, the GaussianNB and KNeighbors algorithms fared well, but their performances are not taken into account when compared to the performances of the Random-Forest and Decision-Tree methods. The Random-Forest method was the most effective when comparing the two top algorithms in the study in terms of performance. The Random-Forest approach was considered better across all metrics used in the analysis. Although it was not the finest, the Decision-Tree was also reliable. A consistent network of many (n estimators can be set) connected decision trees makes up the Random-Forest ensemble machine learning method. The Random-Forest technique is trained using samples drawn at random from the training data frame with replacement. In doing so, it creates a set of decision trees with deliberate variations [38,39]. The ultimate output or forecast is thought of as the output that is obtained by the majority of decision trees. Each decision tree has its own result. As a result, the Random-Forest technique makes effective use of a labelled training dataset. This explains why it predicts all ventilation modes with the maximum accuracy across all parameters. As a result, an approach for creating a data frame pertaining to a work has been offered in the current study effort [36]. The best feature selection test produced the final data frame, which contained five significant features. A comparative analysis has identified the best ML algorithm for predicting the ventilator mode setting on a per-breath basis. In this study, all three categories of distinct modes were examined. Based on the inputs it was given, the built ML model projected that one of the five ventilation modes—pressure control (PC), volume control (VC), pressure support (PS), proportional aided ventilation (PAV) and continuous positive airway pressure (CPAP) modes—would be the output. The accuracy of the predictions made by the Random-Forest ML model for each mode of ventilation was quite high. The average accuracy of the Random-Forest ML model was 0.9889, which was higher than that of other studies [34,35].

## 4. Conclusions

Mechanical ventilation therapy necessitates a thorough data analysis before deciding on the appropriate ventilation strategy for a particular patient. In the current study, the per breath dataset of the chosen features is first checked for statistical correctness using descriptive statistics, the data’s reliability is assessed using Cronbach’s Alpha (0.787), and finally it is used to train the machine learning algorithms. The results of the comparative study show that the best ML technique for predicting the right ventilation mode setting is a Random-Forest algorithm with precision ranging between 0.98 and 0.99, recall ranging between 0.98 and 1.0 and an F1 score ranging between 0.98 and 0.99. However, this study was subject to a few limitations. The data needed to create the ML model could only be obtained from one healthcare facility. The importance of the ML model for ventilation mode prediction will increase if it incorporates data from diverse international healthcare facilities. Furthermore, only five modes were investigated in this study; however, additional special modes, such as pressure-regulated volume control (PRVC) modes and synchronized intermittent mandatory ventilation (SIMV), must be investigated in future research. As a result, for further advancements in ventilation mode prediction using ML techniques, the Random-Forest method may be a good choice with the addition of more diverse and global data.

## Figures and Tables

**Table 1:** Classification of modes based on IPPV principle.

Traditional Modes	Advanced Modes	Biphasic Modes
Continuous mandatory ventilation (CMV) Synchronized intermittent mandatory ventilation (SIMV) Support mode	Proportional assist ventilation (PAV) Proportional pressure support (PPS) Adaptive support ventilation (ASV) Automatic controller of oxygen Neutrally adjusted ventilatory assist IntelliVent SmartCare	Continuous positive airway pressure (CPAP) DuoPAP mode Airway pressure release ventilation (APRV)

**Table 2:** Control parameters to be set in a mode.

Controls for Ventilation	Controls for Adequate Oxygenation	Controls for Patient–Ventilator Synchronization
Inspiratory Tidal Volume [VTi (ml)]	Fraction of inspired oxygenation FiO_2_ (%)	Trigger sensitivity
Mandatory breaths per minute [ f (rpm)]	Positive end expiratory pressure	Patient trigger (pressure or flow)
Inspiratory time (s) I:E ratio (inspiratory to expiratory time ratio) Pressure control (cm H_2_O) Pressure support (cm H_2_O)	[PEEP (cm H_2_O)]	Flow cycle

**Table 3:** Descriptive statistics of data.

N	Minimum	Maximum	Mean	Std. Deviation	Variance	Skewness		Kurtosis	
Statistic	Statistic	Statistic	Statistic	Statistic	Statistic	Statistic	Std.Error	Statistic	Std.Error
20,237	61.00	700.00	390.6240	122.65019	15,043.069	0.421	0.019	0.104	0.038
20,237	1.00	1170.00	396.7983	133.21416	17,746.013	0.164	0.019	0.873	0.038
20,237	0.23	14.51	7.4352	3.05062	9.306	0.666	0.019	1.067	0.038
20,237	0.15	36.80	11.5536	7.29206	53.174	0.805	0.019	0.736	0.038
20,237	0.28	14.51	7.4459	3.05173	9.313	0.664	0.019	1.081	0.038

**Table 4:** Reliability statistics for data.

Reliability Statistics			Hotelling’s T-Squared Test				
Cronbach’s Alpha	Cronbach’s Alpha Based on Standardized Items	N of Items	Hotelling’s T-Squared	F	df1	df2	Sig
0.787	0.89	5	168,373.607	42,085.629	4	20,237	0

**Table 5:** Pearson correlations.

	TVi	TVe	PEEPprev	Pmin	PEEP
TVi	1	0.174 **	0.095 **	0.272 **	0.014
TVe	0.174 **	1	0.287 **	0.039	0.403 **
PEEPprev	0.095 **	0.287 **	1	0.502 **	0.035
Pmin	0.272 **	0.039	0.502 **	1	0.082 **
PEEP	0.014	0.403 **	0.035	0.082 **	1

** Correlation is significant at the 0.01 level (2-tailed).

**Table 6:** Parameters tuned in the ML algorithms.

ML Algorithm	Parameters	Definition	Value
Random-Forest	criterion	Quality measurement of a split.	Gini
	n_estimators	The number of trees in the forest	0–100
Logistic Regression	tol	Tolerance for stopping criteria.	10^−4^
	C_inverse	Inverse of regularization strength.	1.0
	max_iter	Max iterations taken for solvers to converge.	100
Support Vector Machine	penalty	Specifies the norm used in penalization.	l2 1000
	max_iter	Max number of iterations to be run.	
	C	Regularization parameter.	
Guassian Naive Bayes	var_smoothing	Portion of largest variance including	0.1 10^−9^
		all features added to variances for	
		stability calculation.	
K Nearest Neighbors	n_neighbors	Number of neighbors to be used. Weight function to be used. Algorithm used to compute the nearest neighbors.	5 uniform auto
	weights		
	algorithm		
Decision-Tree	criterion	Quality measurement of a split. Strategy used to choose the split at each node.	Gini best
	splitter		

**Table 7:** Model evaluation report for Random-Forest algorithm.

Modes	Precision	Recall			F1-Score
		Training	Testing	Overall	
CPAP	0.99	0.99	0.98	0.99	0.99
PAV	0.98	0.97	0.99	0.98	0.98
PC	0.99	0.99	1.0	1.0	0.99
PS	0.98	0.97	0.99	0.98	0.98
VC	0.99	0.99	0.98	0.99	0.99

**Table 8:** Model evaluation report for Logistic-Regression algorithm.

Modes	Precision	Recall			F1-Score
		Training	Testing	Overall	
CPAP	0.17	0.09	0.11	0.10	0.15
PAV	0.33	0.11	0.10	0.11	0.17
PC	0.67	0.82	0.85	0.84	0.75
PS	0.59	0.99	0.98	0.98	0.73
VC	0.90	0.71	0.69	0.70	0.79

**Table 9:** Model evaluation report for GaussianNB algorithm.

Modes	Precision	Recall			F1-Score
		Training	Testing	Overall	
CPAP	0.53	0.87	0.88	0.88	0.66
PAV	0.41	0.36	0.39	0.38	0.39
PC	0.79	0.77	0.79	0.78	0.78
PS	0.93	0.96	0.99	0.97	0.95
VC	0.92	0.78	0.79	0.79	0.85

**Table 10:** Model evaluation report for SVM algorithm.

Modes	Precision	Recall			F1-Score
		Training	Testing	Overall	
CPAP	0.73	0.59	0.57	0.58	0.65
PAV	0.63	0.08	0.09	0.09	0.16
PC	0.58	0.95	0.97	0.96	0.72
PS	0.64	0.67	0.69	0.68	0.66
VC	0.97	0.66	0.68	0.67	0.80

**Table 11:** Model evaluation report for KNeighbors algorithm.

Modes	Precision	Recall			F1-Score
		Training	Testing	Overall	
CPAP	0.55	0.61	0.59	0.60	0.58
PAV	0.53	0.51	0.49	0.50	0.51
PC	0.94	0.92	0.89	0.90	0.92
PS	0.73	0.78	0.80	0.79	0.76
VC	0.95	0.96	0.95	0.95	0.95

**Table 12:** Model evaluation report for Decision-Tree algorithm.

Modes	Precision	Recall			F1-Score
		Training	Testing	Overall	
CPAP	0.95	0.94	0.96	0.96	0.95
PAV	0.93	0.91	0.94	0.93	0.93
PC	0.99	0.98	0.99	0.99	0.99
PS	0.97	0.96	0.98	0.97	0.97
VC	0.99	0.99	0.98	0.98	0.98

**Table 13:** Accuracy of each ML algorithm.

ML Algorithm	Accuracy
Random-Forest	0.9889
Logistic regression	0.6790
GaussianNB	0.7804
Support vector machine	0.6819
KNeighbors	0.8165
Decision-Tree	0.9716

## Data Availability

No new data were created or analyzed in this study. Data sharing is not applicable to this article.
